# Current Hospital Topics

**Published:** 1907-04-20

**Authors:** 


### HOSPITAL ADMINISTRATION.

### CONSTRUCTION AND ECONOMICS.

### CURRENT HOSPITAL TOPICS.

**The Waterloo Hospital for Children, S.E.**

This institution has been working for 90 years, and is now being rebuilt as a memorial to her late Majesty Queen Victoria. It has occupied the present site since 1823, and two-thirds of the new hospital have been completed and are now in use. Since March 7, 1899, to the end of 1906, £45,225 have been expended upon land, buildings, and equipment. It is stated that a further £22,700 is required to complete the building and to liquidate the present debt. By special efforts the debt on the building fund has been reduced by upwards of £13,000 during 1906. We consider such a result, under all the circumstances, to be highly creditable to everybody concerned. On page 138 the statement appears in large type that "*the assured annual income is only* £830, *against an estimate of the annual expenditure of* £6,000." On page 16 the report states that the income for maintenance from all sources is £5,836 17s. 1d., while the expenditure is estimated at £4,594. It will be noticed that these statements are in direct conflict the one with the other. The larger figure is made up of annual subscriptions, £1,365; King Edward's Hospital Fund, £500; Hospital Sunday and Saturday Funds, and collections, £153; income from investments, £404; patients' contributions, £297; making a revenue of £2,719, all of which, under good management, may be regarded as assured. It further includes £660 from legacies, the average receipts from this source during the previous five years having been £785, and £2,085 from donations, of which at least one-half may fairly be regarded as assured revenue from this source each year. It thus appears that the total assured revenue at the present time may be set down, under good management, as amounting to at least £4,400 per annum, against an estimated ordinary annual expenditure of £6,000. In view of these facts, we would urge the committee to revise the statements on page 138, for they certainly cannot rest upon the audited accounts, as we have just shown. Nothing does a voluntary hospital more harm, in these days, than to issue an appeal containing figures which do not rest upon a business basis. The larger givers to hospitals properly turn a deaf ear to loose statements, in hospital appeals, which are not supported by audited accounts.

**The Hospital for Diseases of the Skin, Blackfriars.**

This institution has had a somewhat chequered history of more than sixty years. The report, which is stated by the authorities to be out of print, contains certain rules, but they are so loosely drawn that it is not easy to interpret them in a business sense. From the statements contained on pages 5, 6, and 7 of the last report, that for 1905, it seems that donors of £21 qualify as life governors, and an annual subscription of not less than three guineas constitutes a governor. It would appear, however, that both life and ordinary governors have to be proposed for election at a meeting of the committee. There are three trustees of the funds, which amount to upwards of £7,000, and a committee of eight members. At a meeting of the governors which was summoned just before Easter, the committee met—to pass the report—one hour before the governors assembled. After sitting half an hour, we are informed by one of those present, that the door was opened and the assistant-surgeon, with a few other persons, raided the room, and made themselves very objectionable to some of the committee present, including the chairman. They then constituted themselves a meeting of governors, though the hour fixed for the annual meeting had not yet arrived, and proceeded without more ado, though they would appear to have had no locus for the purpose, to elect themselves a committee, and to appoint the assistant-surgeon as full surgeon and chairman of the Board. How irregular these proceedings were will be manifest from the fact that, the assistant-surgeon has only held office for two years, whereas rule 8 provides no assistant is eligible to be elected surgeon before he has served six years as assistant, unless special circumstances, in the opinion of the committee, require the waiving of this rule in favour of a particular individual. Here, then, we appear to have the case of a so-called hospital without proper regulations, which has been raided by a few individuals, who claim the right to take possession and rule the institution, without apparently legal status or authority. It seems to us, in such circumstances, that the old committee is the only body with authority to govern the institution. We expect that the trustees will move the courts to protect the subscribers' interests, by authorising them to carry on the affairs of the institution in accordance with its rules, until such time as they can reorganise the whole hospital and place it on a business basis. Neither King Edward's Hospital Fund nor the Hospital Sunday Fund makes any grant to this hospital, and we should hope that the Hospital Saturday Fund authorities will take prompt steps, to mark their sense of the existing chaos, by refusing to contribute anything further to the funds, until the whole institution has been reconstituted and reduced to order. As matters stand at present a grave scandal appears to have been created, and we presume that that is the view which is likely to be taken of the recent proceedings by the advisers of Her Majesty Queen Alexandra, whose name appears as patroness, and by the vice-patron the Duke of Rutland. In view of the facts we have stated, can anyone be surprised that in the present day certain special hospitals do not commend themselves to public countenance or public support?

